# Additive Manufacturing Processes in Selected Corrosion Resistant Materials: A State of Knowledge Review

**DOI:** 10.3390/ma16051893

**Published:** 2023-02-24

**Authors:** Alisiya Biserova-Tahchieva, Maria V. Biezma-Moraleda, Núria Llorca-Isern, Judith Gonzalez-Lavin, Paul Linhardt

**Affiliations:** 1 Departament Ciència de Materials i Química Física, Universitat de Barcelona, 08028 Barcelona, Spain; 2 Departamento Ciencia e Ingeniería del Terreno y de los Materiales, Universidad de Cantabria, 39004 Santander, Spain; 3 Institute of Chemical Technologies and Analytics, Vienna University of Technology (Technische Universität Wien), 1040 Vienna, Austria

**Keywords:** corrosion, additive manufacturing (AM), titanium alloys, aluminum alloys, duplex stainless steels, defects

## Abstract

**Highlights:**

- A full review article on the corrosion behaviour of additive manufacturing (AM) of metallic parts is presented with the aim of covering the significant lack of information on this subject.
- Corrosion resistance of light metallic systems and duplex stainless steels objects obtained by additive manufacturing (AM) processes is critically discussed, based on the synergism between variables linked with chemical composition, manufacturing processes, and service conditions.
- Methodologies for improving the corrosion resistance of metallic parts produced by additive manufacturing (AM) are highlighted.
- The available data allow us to state that potentiodynamic corrosion tests are the best methodology to characterize metal additive manufacturing (MAM) corrosion and this unique test should be selected for the evaluation of this phenomenon. There is a gap in knowledge and test procedure to properly identify the relationship between defects and corrosion behaviour of the most studied metal additive manufacturing (MAM) systems.

**Abstract:**

Additive manufacturing is an important and promising process of manufacturing due to its increasing demand in all industrial sectors, with special relevance in those related to metallic components since it permits the lightening of structures, producing complex geometries with a minimum waste of material. There are different techniques involved in additive manufacturing that must be carefully selected according to the chemical composition of the material and the final requirements. There is a large amount of research devoted to the technical development and the mechanical properties of the final components; however, not much attention has been paid yet to the corrosion behaviour in different service conditions. The aim of this paper is to deeply analyze the interaction between the chemical composition of different metallic alloys, the additive manufacturing processing, and their corrosion behaviour, determining the effects of the main microstructural features and defects associated with these specific processes, such as grain size, segregation, and porosity, among others. The corrosion resistance of the most-used systems obtained by additive manufacturing (AM) such as aluminum alloys, titanium alloys, and duplex stainless steels is analyzed to provide knowledge that can be a platform to create new ideas for materials manufacturing. Some conclusions and future guidelines for establishing good practices related to corrosion tests are proposed.

## 1. Introduction

AM is continuously growing and helping to expand the options for materials used in manufacturing. Although thermoplastics have been the most attractive materials in the early days of 3D printing, metallic systems have been driving an important revolutionary change in AM during the last decade, addressing fundamental challenges for engineers and researchers.

AM has opened a huge new research field and prompted the investigation of alloys and new techniques in order to optimize the whole process, from the raw material properties to the final component characterization. This is due to the advantages that the process can provide compared to the traditional manufacturing techniques. Additive manufacturing development is exponentially accelerating cost adjustment and efficiency in producing different components for a wide range of applications. The reduction in energy of up to 25% and cutting of waste and materials costs up to 90% are some impactful achievements, in addition to the main attractiveness of the AM processes.

Furthermore, a variety of complicated shapes can be easily designed through the AM technique and, thus, specific components can be obtained for biomedical applications [1,2,3] and the aeronautical industry [4,5,6], with titanium and aluminum alloys being, respectively, the most-used metallic materials in such applications due to their mechanical properties and lightness which are essential to both industries. Fe-based materials, and more specifically, stainless steels, as the best substitutes in applications where corrosion resistance in combination with mechanical properties is required, are frequently used in AM, as well. Understanding the AM process conditions, postprocessing, and structure-performance of stainless steels should be considered to create more corrosion-resistant stainless steels [7,8]. There is also growing interest in the use of AM for the production of jewelry and luxury watch components, as well. This interest is driven not only by the potential design innovation offered by Additive Manufacturing, but also from the environmental and economic point of view, as the recovery of precious metals such as gold is a complex process, whereas this step is circumvented by applying gold as an alloying element. The main precious metal that benefits nowadays from AM is platinum and its alloys, which are used to create highly-value added metallic objects [9]. Nickel-aluminum bronze (NAB) alloys are extensively used in the marine industry and there has been a recent increase in interest in their being additively manufactured. Their microstructure can vary in comparison to conventionally casted NAB alloys and some studies have shown improvements in their mechanical and corrosion properties [10].

Such applications require high-quality control and specific corrosion resistance properties besides mechanical resistance. The influence of the conventional techniques on such properties in metallic components produced by AM has been extensively studied. However, it has been observed in the literature that there is a lack of information and data, specifically on the corrosion properties of AM metallic materials in comparison to traditionally fabricated objects. For these reasons, the main purpose of this paper is to obtain a state of the art of the most significant problems related to all kinds of defects occurring during and after the main AM processes and to correlate these issues to the corrosion resistance of the as-built AM metallic parts.

## 2. Additive Manufacturing Processes Classification

### 2.1. Introduction

In order to understand the impact of additive manufacturing processes, a general definition and description, as well as classification of the various additive manufacturing processes, will be covered in the following section.

Additive manufacturing (AM) is defined by the ISO/ASTM 52900 [11] as the process of joining materials to make objects from 3D model data, usually layer upon layer, as opposed to subtractive manufacturing methodologies, such as the traditional machining of building components. AM refers to a variety of processes in which material is deposited, joined, or solidified [12]. Metal additive manufacturing (MAM), also known as metal 3D printing, consists of complex metal parts fabrication with improved functionalities. The advantages of using AM of metals over traditional manufacturing methods, in some cases, have led to an enormous increase in its use; for example, between 2020 and 2024, metal AM growth is estimated to be around 14% per year [13], alongside a simultaneous increase in the number of studies on additively manufactured steels. For instance, 5.861 articles were found in the Web of Science, considering both additive manufacturing and steels, that were published in the last five years. Since its inception in 1987, AM has shown significant progress in the general understanding of the processes related to it and the impact on the structures and properties of the fabricated metallic components.

AM technologies are based on the principle of modelling a body, loading the data into the equipment, and building the component layer upon layer until the creation of the complex 3D model. AM can be categorized according to the energy source provided during the process (e.g., laser or electron beam). 3D printing deposition is carried out by means of a print head or a nozzle (or multiple) that deposits the material in a controlled flow and temperature.

This layer-by-layer forming saves costs because it does not generate large amounts of waste, as the material is only added where it is really needed, thereby permitting near-net shape manufacture with powder or wire form as the starting feedstock. Virtually, this process does not create any residual material waste if all of the un-melted material can be fully recycled as has been demonstrated recently, focusing attention on the environmental impacts of additive manufacturing vs. traditional machining via life-cycle assessment in a comparative way [14]. Also, almost any geometry can be created, in contrast to conventional machining, the walls of the workpiece can be very thin (which is not possible through casting), and manufacturing is possible in a fairly short period of time. The freedom of design is particularly relevant in biomedicine, as dental implants or orthopedic parts and can be adapted to the specific needs of each patient [15]. However, the main sector driving the increase in the use and development of AM is the automotive sector, due to reductions in weight and, especially, in the weight/cost ratio.

In any case, we must not forget that there are also problems associated with this general technique; therefore, it is essential to study these issues in order to understand and solve them, with the aim of obtaining an even more versatile technique. To this end, numerous studies have been carried out comparing the mechanical properties, corrosion behaviour, and microstructure of metal parts produced by AM and by traditional manufacturing methods [16,17]. It is known that the special conditions of AM produce a fine microstructure with unique directional growth characteristics in non-equilibrium zones. This distinctive microstructure, fine α′ martensite in as-built Ti6AlV4 [18,19], together with microstructural defects, originated from the additive manufacturing process, strongly influences the corrosion behavior of metal additive manufacturing materials. Several studies have already been carried out in this respect. However, issues concerning the corrosion and corrosion protection of these materials are still poorly understood [20].

Some AM processes have evolved from conventional welding processes, while others, such as powder bed fusion processes, have been developed with the specific intent of enabling the manufacturing of complex 3D geometrical objects. Two main groups can be distinguished in this technique:

- Powder bed technologies. Within this group, a classification can also be made according to the energy source used for deposition. Here, we find Selective Laser Melting (SLM), Electron Beam Melting (EBM), or precision inkjet printing in which the metal powder is mixed with a binder, so that after deposition the piece is sintered, resulting in the final model.
- Blown powder technologies, also known as Laser Metal Deposition, LMD, or Laser cladding, wherein both are based on the availability of the metal powder to blow coaxially to the laser beam, which melts the powder onto a metal substrate to form a metallurgical bond upon cooling to room temperature.

Depending on the material used, a distinction can be made between metal in the form of ‘wire’ and metal in the form of ‘powder.’ For the former, the metal is placed in the form of a coil which is connected to the extruder nozzle. For the latter, several requirements must be met. For additive manufacturing, metal powder should have a spherical shape to ensure good flow and coating ability. A particle size range between 50 μm and 150 μm, depending on the machine type and its distribution, must be adapted to the application, as well as the chemical composition of the metal and the gas content, because these may be responsible for defects within the structure [21,22].

There are also other causes associated with AM processes that can produce defects in the structure. One example is the thermal cycling inherent to manufacturing, resulting in different microstructures and types of defects related to the process parameters and the geometry of the final object, as well as the local environmental conditions during processing.

If the manufactured part has a low static loading, the microstructure itself can determine the average mechanical properties. However, if it must withstand cyclic loading, as in aero engine or turbine components, defects limit the lower threshold of the mechanical properties and, therefore, are a major concern, as they restrict the loading conditions during operation. In view of the above, post-manufacturing treatments such as Hot Isostatic Pressing (HIP) are essential, as they can minimize certain types of defects, such as porosity, depending on the material and the AM process used. Other treatments include hybrid solutions between in-situ and post-manufacturing treatments, such as the induction of compressive residual stresses in the material to reduce the influence of surface topography, defects, and residual stresses.

Sander et al. [23], in 2018, published a deep, extended, and critical review of corrosion knowledge of Additively Manufactured alloys, pointing out that this revision practice should be done each year, since AM is being introduced in all industrial sectors in which severe and critical service conditions are present. Indeed, this is the motivation of this paper: to advance the corrosion behavior knowledge of the AM metallic systems, paying special attention to the deep relationship between the existence of defects resulting from each particular process as well as the final microstructure of the produced material. The following sections present a description and classification of the main AM methods and tools that are currently carried out to produce metallic components.

### 2.2. Additive Metallurgy by Direct Energy Deposition Processes (DED)

During direct energy deposition, focused thermal energy is used to melt the material as it is deposited. This is the main difference of Powder Bed Fusion (PBF), where thermal energy is used to selectively fuse regions of a powder bed. DED processes are typically used on existing parts of arbitrary geometry with a relatively high deposition rate; however, they allow for little complexity in shape. Therefore, their main use is in the repair or improvement of other pre-formed parts.

#### 2.2.1. Additive Metallurgy by Laser Melting Deposition (LMD)

The main advantage of this method is the lack of Heat-Affected Zones (HAZ), or hydrogen cracking, which is characteristic of repairs by conventional arc welding, or cold or plasma spraying. In fact, LMD can reduce distortion and microcrack formation by optimizing the parameters, resulting in metallurgical-bonded and high-quality coatings obtained in a short period of time. When used to repair parts, it is done with highly satisfactory results. With respect to the disadvantages, a typical example is when LMD is used with 316 L stainless steel, in which the composition has a great effect, for instance with any redistribution of Mo in printed 316 L alloys. If this happens, their corrosion resistance could be significantly affected, as well as the grain size, altering local degradation mechanisms which have a strong dependence on the corrosive media (decreasing grain size in austenitic-type 304 stainless steel below 2 µm increases the general corrosion in sulfuric acid solution due to passive film destabilization at grain boundaries) [24,25].

#### 2.2.2. Additive Metallurgy by Wire Arc Additive Manufacturing Processes (WAAM)

**Figure 1:**
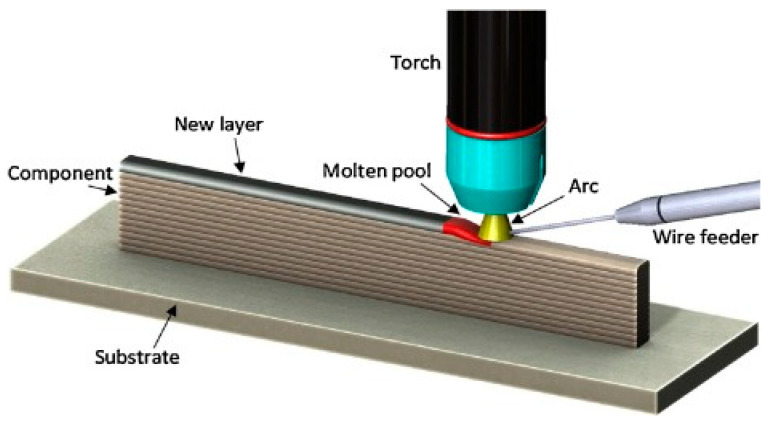
Illustrative diagram of WAAM process and Schematic representation of different zones in WAAM part, being MPB: melt pool border, MPZ: melt pool zone, and RM: remelted, HAZ: heat affected zone [27].

WAAM uses arc welding tools and makes use of wire as feedstock for additive manufacturing. The deposition rate is high, the equipment is not expensive, and it has a good structural integrity, making it a promising method to replace the current manufacturing methods for components with low and medium complexity [23]. During the WAAM process, every single layer has a heterogeneous grain structure (as well as melt pool boundaries, matrix supersaturation, segregation, phase transformation, new textures, and oxide formation), as in other types of MAM; however, in this case, the former layer is in a solid-state, and, therefore, holds a lower temperature, increasing the effect. In addition, for each individual layer, three zones can be distinguished (Figure 1): the Melt Pool Zone (MPZ), Melt Pool Border (MPB), and heat affected zone (HAZ). The MPZ is produced because the metal wires are completely melted during WAAM. Between different MPZ areas, it is possible to find the MPB, with a completely different grain structure and intermetallic distribution. Finally, HAZ is the previously deposited layer region where the temperature is high enough to alter the microstructure but below liquidus temperature. The region which is the furthest away is total or partially remelted, and belongs to the MPB on the previously deposited layer side [26].

### 2.3. Additive Metallurgy by Powder Bed Fusion Processes (PBF)

Different scan parameters should be considered when the PBF process is carried out. An inert atmosphere or partial vacuum is necessary to provide shielding of the molten metal. Currently, the most-used practice is laser powder-bed fusion (L-PBF) followed by vacuum heat treatment to produce alloys with controlled microstructures. An energy source is used to selectively melt each layer of the powder, which are already spread according to the required cross-section of the part in the digital model. Once the layer has been scanned, the piston in the building chamber moves down and the piston in the powder chamber moves up according to the defined layer thickness. The coating mechanism or roller deposits the powder in the building chamber, which is scanned again by the energy source. This cycle is repeated layer by layer until the complete part is formed. The time required to complete a part is longer than that for DED technologies; however, a higher complexity and better surface finish can be achieved and minimal post-processing is required. Multiple parts can be built together to make maximum use of the build chamber [28]. If the process is carried out at reduced pressure, a more stable melt pool and reduced porosity are obtained. Part-specific process settings are often controlled to reduce thermally-induced residual stresses and defects.

#### 2.3.1. Additive Metallurgy by Laser Powder Bed Fusion (L-PBF)

Powder-Bed category techniques involve laser powder bed fusion (L-PBF), also known as selective laser melting; SLM; selective laser sintering, SLS; and EBM technologies. The cooling rate for SLM is usually >105 K/s, which is higher than that of direct laser deposition (DLD) (usually from 103 to 105 K/s) and much higher than that of traditional casting methods, which have lower solidification rates [29]. The local rapid heating and, especially, fast cooling rates, coupled with thermal cycling, induce the formation of unique microstructures with refined grain structures, dislocation cells, and internal residual stresses. These conditions also cause the formation of metallurgical defects, including un-melted powder particle microcracks, entrapped gas pores, balling, and rough surfaces.

One of the most common nucleation sites for corrosion problems in three-dimensional printed materials is pores. They reduce the passivation property in the presence of sulphuric and phosphoric acid solutions [30]. There are two types of pores: one type exists around the un-melted powder particles, and another is caused by the trapped gas inside the powder during gas atomization [31]. The porosity can be reduced to a certain extent by optimizing the printing conditions, including laser energy, scanning rate, and scanning direction. It has been found that increasing the laser power or properly decreasing the scanning rate can reduce the porosity of different metals such as nickel- or aluminium-based alloys, or some type of stainless steel such as 316 L by SLM [23]. This will be detailed in the next section.

Selective laser sintering, SLS, was the first PBF-AM process, invented in 1981 by Ross Householder [32] who patented it under the name of “Molding process”. A few years later, Carl Deckard and Joe Beaman patented a similar method, under sponsorship of DARPA [33], which was more alike to what is known today as SLS.; however, it would take some years before this technique could be used with metallic materials, as it was initially used with amorphous or semi-crystalline polymer powder or semi-crystalline powder. Usually, this technique is related to a final step that consists of applying thermal treatment in order to remove the polymer binding particles, sintering, and improving the base material microstructure [34,35,36].

In any case, there are examples of the strength of this technique, such as the seven degree-of-freedom dual-arm hydraulic which was built using the SLM method for undersea use. The hydraulic system was produced with titanium to obtain the necessary properties for naval applications [37].

#### 2.3.2. Additive Metallurgy by Electron Beam Melting (EBM)

Many parameters can be modified and corrected using the EBM technique so that the final part does not require further treatments. Precursor and pre-alloyed powders are selectively preheated and melted by varying the beam sweep, its current, and even the cooling rate, this being one of the main causes of defects in AM due to creating stresses in the microstructure. If the microstructure and mechanical properties of these systems are analyzed and compared to conventional forged and cast products, a columnar microstructure resulting from layer-by-layer melting solidification phenomena can be distinguished. This is because solidification is directional, which implies a continuous melting/solidification front [38].

Some of the major defects that appear during this process are unavoidable to some extent and can severely degrade the mechanical properties of the material. An example of this is Alloy 718, obtained by EBM, which is often post-processed to improve the material properties. Although hot isostatic pressing (HIP) is commonly used to close defects, it cannot close open-to-surface ones. Therefore, a possible solution could be that, if the surface of the EBM-manufactured specimen is suitably coated to encapsulate the EBM-manufactured specimen, then HIPing can be effective in healing such surface-connected defects [39]. It is remarkable that the residual properties of EBM-fabricated components are usually as good as or better than conventional cast or wrought products, even after post processing. Table 1 summarizes the main advantages and disadvantages of both the DED and PBF AM techniques.

### 2.4. Additive Metallurgy by Hybrid-AM Techniques

**Figure 2:**
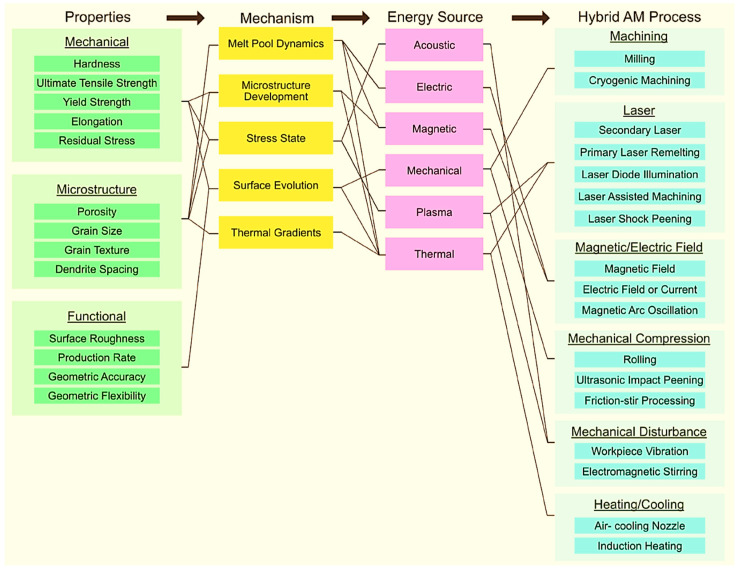
Property—mechanism—energy source—hybrid-AM process framework for hybrid-AM classified by mechanism and energy source utilization. Attending to the scheme, it is easy to understand why such a problem-oriented selection of the sample is possible [46].

As already mentioned, AM processes offer numerous advantages, especially in terms of free design in a reduced time; however, this is irrelevant if the part created is full of defects that can affect the mechanical, physical, or chemical properties in such a way as to render the part unusable. To avoid this, in situ or serial AM processes and secondary energy sources that are capable of modifying the resulting material or part properties must be combined. These modifications can be due to both in-situ secondary procedures and process chains, and is anchored in multiphysics mechanisms, so that new hybrid-AM processes can be applied very selectively to the problem which was generated during AM (Figure 2). The desired material properties determine the mechanism that is used and, in turn, the energy source that is applied, which ultimately defines the hybrid-AM process. Some of the properties that can be changed using this mechanism are melt pool dynamics, microstructure development, stress state, surface evolution, and thermal gradients [46].

Overall, additive manufacturing processes for metal systems promise significant revolution in the industry, assuring advantages but also many challenges that must be faced in the upcoming century.

## 3. Defects in Metallic Systems Due to AM Processes

The control of defects in all processing of metals is of crucial importance, and in the same is true of additive manufacturing processes, as these can significantly deteriorate mechanical properties. Additionally, it is even more important to minimize or prevent processing defects when corrosion resistance is required from the processed components. Therefore, in this section, we will discuss the appearance of different defects during the processing of metals and their influence on the loss of corrosion properties.

Despite the great advances and improvements provided by AM techniques, as has already been mentioned in the previous section, the appearance of defects in the manufactured parts can significantly worsen the physico-chemical properties of the components and, with this, their functionality. Since a defect is a discontinuity of a material that penalizes the properties of that material, they must be avoided during the processing of metallic systems. The key issue is to define the concept of defect in the material processed by additive manufacturing due to the particularities of each process concerning the material to be processed, such as chemical composition and geometry, as well as the variables of the process: temperature, flux heat speed, vacuum system, etc. Therefore, it is highly dependent on the process itself.

Defect formation is a common problem in all types of AM, being commonly identified in WAAM and SLM; nevertheless, the defects generated during WAAM are the most studied [47], wherein residual stresses and distortion parameters, among others, can influence the fracture behavior and as a result it can be difficult to achieve the required tolerances of the components. In this sense, a defect is a discontinuity; however, in general, larger and more heterogeneously distributed defects would imply a higher negative impact related to the same situation in other classical processing methodologies. In this way, the critical defect criteria, which are type, size, orientation, and distribution, may be known. Hence, it is also important to understand the properties of AM components and the associated effects of defects, mainly porosity and cracks, to understand their impact. Previous studies have defined the term as critical defect criteria [48].

**Figure 3:**
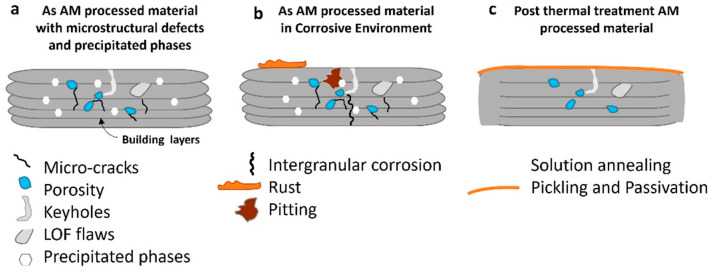
(**a**) Schematic representation of an AM-processed material and the presence of several microstructural defects (micro-cracks, porosity, keyholes, and LOF flows, as represented); (**b**) the same AM processed material in a corrosive environment where intergranular corrosion, pitting corrosion, and rust on the surface can appear; (**c**) the same AM-processed material after being thermally treated (solution annealing) to remove residual stresses and precipitated secondary phases after pickling and passivation as a preventive measure for corrosive environments.

Corrosion could be controlled by the existence of defects that are caused during the additive manufacturing process; therefore, it is important to know the defects present in a material processed by AM and their influence on the final properties. In this section, the description of the main defects found in the literature and their direct correlation to the properties of several components of alloys obtained by the AM process will be shown. The main defects are not strictly categorized by a unique type; nevertheless, the most frequently found are cracks, porosity, residual stresses, non-homogenous microstructure, unbalanced chemical composition, brittle phases nucleation, properties anisotropy, and microgalvanic cells. Some of these defects are shown in the schematic illustration in Figure 3a, which represents their formation during the layer-by-layer building procedure of AM processes; for example: porosity, lack of flow parts, keyhole defects, microcracks, and the formation of intermetallic phases that are not visible macroscopically (not represented in the scheme). Figure 3b details the appearance of additional defects due to the influence of a corrosive environment on the as-built AM component. For instance, intragranular corrosion and pitting corrosion defects could be present in the microstructure along with the AM process defects, causing significant microstructural damage. Figure 3c offer a general view of a component after post AM processes such as solution annealing treatment at a certain temperature and during a specific time, as well as the surface pickling passivation process, which improves the homogenization of the microstructure and protects, superficially and internally, the material from corrosive environments, respectively.

Wu et al. [47] have published a state of the art focused on the defects found in different alloys used in WAAM in order to control the quality improvement requirements for specific industrial applications. An algorithm has been optimized to identify the correct process parameters by Busachi et al. [49] in order to identify the main defects generated during the WAAM process: porosity, undercutting, and humping. B. Zhang et al. [50] reviewed defect formation mechanisms and their classification in SLM for common defects such as porosity, incomplete fusion holes, cracks, surface roughness, and delamination. However, it is necessary to point out that the type of defects is very closely dependent on the chemical composition of the material to be processed, as has been observed by Xu et al. [51] in AlSi10Mg alloy used in SLM, with lack of fusion and porosity being identified as the most common defects. Residual stresses are usually induced in SLM process due to high thermal gradients occurring in the process that can also cause cracks and distortions in the manufactured material, as has been observed by Siddique et al. [52].

Sander et al. [23] conducted an exhaustive state of the art review, linking the chemical composition with the corrosion behavior of numerous materials in multiple adverse conditions. They have pointed out the necessity of a deeper fundamental understanding of MAM and have called attention to the necessity of standards and standardized assays, which can be, for example, specimen preparation. Actually, it is critical to approach an unified understanding of the complex relations between all of the variables involved in each process. Nevertheless, the increasing development of additive manufacturing techniques as an alternative to conventional processing, having an impact on new metallic materials, needs to proceed with comprehensive periodical revision, which is one of the main purposes of this paper.

### Classification and Identification

Since the different processes can induce different defects, it is not easy to establish a close classification of them. Moreover, some hypothetical defects could be considered as desirable variables during an AM process, such as porosity, in order to produce lighter components despite the fact that they are cavities within the weld bead. In particular, its presence reduces the overall density of the parts and thus reduces the mechanical properties of the manufactured components. In this study, we will only be considering defects which introduce a penalizing issue to the material microstructure and/or macrostructure. The formation of keyholes is the main defect that appears in WAAM and SLM build components [53]. In research conducted by the LLNL [54], a previously unknown dynamic within LPBF was revealed, in which the process produces small particles or clusters of powder particles that are ejected from the laser’s path and which can land back on the parts. This phenomenon can lead to the formation of pores or defects in final parts. After discovering these spatter interactions, LLNL researchers set out to better understand them and find a solution for reducing or even eliminating defects in parts built through a common, laser-based metal 3D-printing process. They show a simulation of a laser interaction, where laser-power was above a threshold that expelled the spatter away from the scan track, preventing the formation of defects due to “laser shadowing”.

Porosity refers to cavities within the weld bead and is considered a defect as it affects the performance of the weld. In particular, its presence reduces the overall density of the parts and thus reduces the mechanical properties of the manufactured components. Undercutting is related to the concavity of the weld bead, which compromises the tolerance requirements. Humping refers to an uneven material deposition. LOF defects are the result of an interaction of spatter particles of the material with the incident laser beam. Kim et al. [39] classified two porosity types in AM-produced parts: lack-of-fusion (LOF) porosity, resulting from the interaction of spatter particles of the material with the incident laser beam, and gas porosity, (GP). LOF does not necessarily come from the interaction of spatter particles but can also result from an insufficient energy input which does not completely melt the powder bed.

The various types of microstructural features or defects, their generation mechanisms, their effect on bulk properties, and the capability of existing characterization methodologies for powder-based AM parts were considered by Collins et al. [55], who pointed out the importance of their in-situ detection by non-destructive tests. Recently, Snow et al. [56] found that these flaws in AM-produced material are an important defect that penalize fatigue resistance, by the application of machine learning approaches they distinguish different types by attending to geometry and size: gas porosity and keyhole pores, (~50 μm or less) relatively spherical, and lack of fusion pores with an irregular morphology, often much larger,. Nassar et al. [57] showed that stochastic collisions occur both between particles which are nearly-simultaneously expelled from the laser interaction zone and between particles which are ejected from distant locations to support the existence of LOF defects. The effect of ejected melt and its spatter in PBFAM specimens has been studied, the occurrence of cracks being the main associated defect. Monzon [58] defined three methods to check porosity and determined the strong relevance of this defect on fatigue crack patterns in SLM Ti-6Al-4V alloys.

Since the microstructure is fairly anisotropic in AM components, and an extensive columnar zone can penalize mechanical properties, the research carried out by Blinn et al. [59] in which they claimed the possibility to control the full microstructure of an AISI 316 stainless steel, avoiding potential posterior defects, is interesting. By modulating spatial laser intensity profiles on the fly, site-specific microstructures and properties can be directly engineered into additively manufactured parts, as has been shown by Roehling et al. [60].

Porosity is very critical, in particular for aluminum alloys, as has been studied recently by several authors [61,62] and has permitted the establishment of a classification of small and homogenously distributed hydrogen pores and big and inhomogeneously distributed process pores. Hauser et al. [63] have observed the formation of aluminium oxide wherein the oxygen source is both from the wire and the substrate, which dissociates in the arc-based process and could enter the melt pool, causing a high number of pores in aluminum alloys, this seems to be related to the high shielding gas flow rate, as well.

Furthermore, porosity plays an important role in NiTi alloys used for biomedical purposes related theirs corrosion behavior since, as porosity increases, higher Ni ion is released compared to the bulk NiTi samples; however, it remains within the range of most of the reported values for conventionally fabricated NiTi alloys [64]. It is worth mentioning that there have been several efforts to reduce the Ni ion release of conventionally-fabricated NiTi parts.

A proper solution annealing heat treatment is recommended for the improvement of the mechanical properties of alloys fabricated by WAAM, as has been observed on Hastelloy C276. It generates a solid solution strengthening, due to a reduction in the brittle phases’ percentages with a PHT at 1177 °C [65]. For a reliable fatigue life, residual stresses must be relieved. The role of residual stresses in this study can be neglected, as a stress-relieving post-process heat treatment was performed on all of the specimens.

The very safety-critical AM components must be inspected after manufacturing to ensure the absence of any concerning defects: either material degradation or deviation from their designed characteristics. The most-used test is based on non-destructive methods, such as the approach phase array ultrasound test to check steel for use in the WAAM process, in which authors generate defects to create a pattern to detect defects [66].

In order to control the existence and distribution of defects, inspection based on acoustic signals in SLM-manufactured parts was proposed by Ye et al. [67]. Meanwhile, Romano et al. [68] proposed X-ray micro-computed tomography (µCT) and metallographic analysis to facilitate the estimation of internal defects of specimens of laser melted AlSi10Mg specimens, demonstrating that CT offers better precision and lower cost than metallographic analysis when similar volumes of material are investigated. In this vein, Nourian-Avval et al. [69] carried out porosity detection in terms of using metallography as computed tomography to study specimens of AlSi12.

Ng et al. [70] showed that there is a clear distinction between two types of porosity in LMD Inconel 718: lack of fusion and gas porosity. Both are the result of different factors which are mainly linked with the process parameters and the melt pool dynamics, and it is possible to optimize their presence in low values. It has also been reported [71] that porosity and irregularities such as molten pool boundary area and columnar structure could be significantly lowered after hot isostatic pressure and ageing after the SLM process are applied to Inconel 718 alloy.

Moreover, it is necessary to point out that no defects have been observed in the interface between the carbon manganese steel and duplex steel FGM when using WAAM and a complete fusion between the two layers [72]. Nevertheless, Bobbio et al. [73] studied graded Ti-6Al-4V to Invar FGM using DED AM, observing that this joint contained morphological defects that included material overflow and macroscopic cracking.

Overall, the main defects detected in conventional processing techniques are also observed in additive manufacturing-processed components. Nevertheless, some defects of AM alloys, may vary in length scale, in comparison to wrought or cast alloys. For instance, porosity or cracking can be from a micron to millimeter scale and residual stress can be on the scale of meters [8]. Table 2 summarizes the main advantages and disadvantages of both DED and PBF AM techniques.

## 4. Light Metals and Additive Manufacturing

Aluminum and titanium alloys are two of the most-studied light alloys due to their excellent corrosive properties, offering at the same time lightness and mechanical properties. Over the last five years, 4.549 articles about titanium in AM were published, according to Web of Science. However, only 1.389 publications investigated the influence of the processing AM on the corrosion resistance. On the other hand, 1.141 articles were published which studied aluminum alloys in AM. Therefore, in this section, general aspects and problems related to AM-processed Al and Ti alloys will be discussed.

Among the materials that can be used with AM techniques are light metals, including beryllium, whose usefulness can be exorbitant within the nuclear fusion sector, thanks to the enormous scope of control provided by AM parameters; however, the main interest in the use of light metals in additive manufacturing comes from industry, especially the automotive, aerospace, aeronautics, and medical industries. This is due to the aforementioned advantages of AM, in addition to the range of possibilities that light metals offer by allow for better controlling of components weight. However, the use of light metals must withstand the harsh conditions to which it must be submitted; therefore, they are not usually used in pure form, but rather as alloys capable of withstanding very severe mechanical loads under critical conditions of pressure, temperature, or corrosive environments, among others.

Thus, developing additive manufacturing process parameters to successfully print these alloys can enhance the adoption of AM in different sectors. All current methods of AM, including but not limited to, laser/electron beam powder beam fusion, blown powder, wire and arc-related techniques, binder jetting, and friction stir processes are of particular interest. This review also focuses on light metals developed through novel AM techniques, which are beyond the current beam technologies.

### 4.1. Main Process and Associated Problems

Some of the more specific problems for these types of materials when used in AM are included in Table 3.

#### 4.1.1. Aluminium Alloys

Aluminium alloys are one of the main systems in which AM is used, especially in the SLM process, and they can be used for a variety of applications, from medical to aerospace. In the latter case, aerospace-grade aluminium is of great interest and is still under investigation [80]. However, processing these alloys is challenging due to the difficulties associated with laser-melting aluminium, where parts may show various types of defects. In recent times, several studies have developed approaches to avoid these defects and reported the successful SLM of various Al alloys [81]. For instance, porosity or high surface roughness, residual stresses, and remaining unmolten powder in the printed parts are some of the defects formed that affect the corrosion resistance properties of Al alloys [82,83,84]. There are several parameters that can influence the final printed parts microstructure and surface roughness, and thus, the corrosion performance. Therefore, post-AM treatments are applicable to reduce such roughness [85,86]. Another example is the application of post-processing electro-polishing, shot peening, or mechanical and electro-polishing, which have shown better corrosion resistance than as-printed parts or sunblasted samples, as reported by Fathi et al. [87].

On the other hand, the effect of the alloying elements such as Si and Mg in AlSi and AlSiMg alloys have shown different corrosion behaviours. Some studies [88,89] have reported that better corrosion behaviour is found in alloys with a higher amount of Si, in comparison to the AlSiMg alloys with a higher amount of Mg. This is mostly due to the formation of Mg_2_Si precipitates that affect pitting corrosion. However, in the as-built AM components, the formation of Mg_2_Si particles have not yet been confirmed. Revilla et al. [20] mentioned many issues in a detailed review about the corrosion resistance of AM Al alloys and have suggested further studies about Mg- and Fe-containing precipitates in Al.

Moreover, solution treatments (T6) on as-built SLM AM parts have been performed in different studies [90,91] to compare the corrosion behaviours within untreated parts. It has been observed that, after exposure to temperatures between 300 and 550 °C for 2 h and water quenching afterwards, separated Si particles in the matrix resulted in higher corrosion densities and lower corrosion potentials. The larger the Si precipitates are, the more restricted is the formation of the compact oxide layer.

A recent review has pointed out the complex and numerous scenarios for cast aluminum alloys and the relationship between microstructure and corrosion resistance [92]. Moreover, aluminum alloys exhibit elongated grains that are aligned with the extrusion direction. This radically changes with treatments leading to thioxoformed high-performance alloy with a regular equiaxial grain microstructure [93].

#### 4.1.2. Titanium Alloys

There are two main reasons why the use of titanium alloys in AM is relevant. The first is the fact that these alloys are biocompatible; therefore, if there is also the possibility to create almost any shape adapted to specific needs, this fabrication is essential. On the other hand, titanium alloys have seen their activity reduced due to their high cost, meaning that it is essential to reduce waste, which is another advantage of AM [94]. Therefore, it seems that the combination of this material with this technique is a great advantage.

In general, it is necessary to properly characterize the mechanical properties of any material to be used; however, due to the medical use of Ti alloys, it is essential to do so after the production by AM. The rapid solidification during the AM process leads to the formation of highly stressed phases, which results in the deterioration of its mechanical properties and the appearance of corrosion on the parts. In addition, Laquai. et al. detected small inhomogeneities such as voids or cracks whose sizes are below the spatial resolution of optical microscopy for Ti-6Al-4V parts produced by selective laser melting [95]. This issue supports the need to use higher resolution techniques to control AM objects.

Zhang et al. [96] presented an extended review of Ti alloys fabricated by the EBM process in which several important issues are explained that are related to the problems within the AM technique, the defects detected afterwards, and the mechanical properties compared to conventionally fabricated components. However, little information is reported about the corrosion resistance properties of AM-fabricated Ti alloys. For instance, they show the results of EBM-fabricated Ti-6Al-4V alloy that exhibits a transformation from the stable prior β phase to stable α phase. Corrosion resistance enhancing has been observed after a higher fraction of β phase and refined lamellar α/β phases with different behaviour compared to traditionally fabricated Ti-6Al-4V alloys [96].

In pure titanium alloys, the microstructure predominately consists of equiaxed prior-β grains, and a body centered cubic crystal structure. As aluminum and vanadium are added, the transformed β phase is presented, which is a varierty of α-morphologies such as widmanstätten; however, vanadium radically alters the microstructure of Ti-Al, since it predominantly leads to a very fine acicular α phase and some are delineated by an intergranular β phase [97].

Chiu et al. [98] have compared the corrosion assessment of Ti-6Al-4V alloy that is conventionally fabricated and a L-PBF build in a body fluid simulation solution. The results showed similar E_corr_ values with a small improvement in the AM-fabricated Ti alloy. Performing a heat treatment adopted before the final application of the as-built AM pieces has been seen as a solution to improve corrosion behaviour. It has been found that the corrosion rate of as-built AM Ti alloys is almost 16 times worse than cold-rolled Ti components, mainly as a result of the non-equilibrium phases formation during AM processes. If the heat treatment is carried out properly, this loss of corrosion property can be reduced or even eliminated. For Ti-6Al-4V alloy produced by the laser-based powder bed fusion AM technique, the ideal process is a post-annealing heat treatment at 800 °C for 2 h, making it so that the corrosion behaviour of the piece can be comparable to the commercial sample ones, due to the stress relief of the martensitic phase and formation of the BCC phase of β Ti-6Al-4V, which has a higher corrosion resistance.

**Figure 4:**
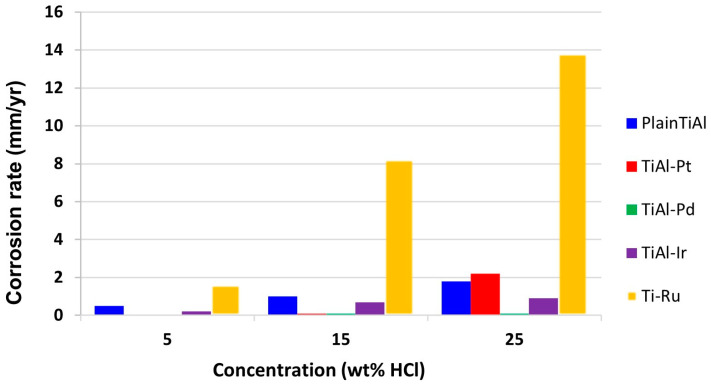
Corrosion rates of Ti-Al alloys with Pt-group metal additions in 5, 15, and 25 wt-%HCl solutions [99].

In the case of Ti-Al alloys, there are three major intermetallic compounds, gamma Ti-Al, alpha 2-Ti 3 Al, and Ti-Al3. Among the three, gamma TiAl has received the most interest and applications. A particular method of improving the stability of the passive layer is adding platinum group metals. Therefore, the corrosion resistance increases, and these alloys are not expected to suffer a relevant corrosive effect, as they increase the corrosion potential to nobler values. Additions of Pt group metals facilitate cathodic depolarisation by providing low hydrogen surge sites, which shifts the alloy potentials in the positive direction, where passivation of the oxide film is possible. Relatively small concentrations of certain precious metals are sufficient to significantly increase the corrosion resistance of titanium in reducing acidic media. A very clear example of how this contributes to corrosion enhancement is in a 25%w HCl solution environment (Figure 4). The addition shifts the cathodic process in the active region of the single Ti-Al alloy. The cathodic modification of Ti-Al alloyed with Pt group metals occurred as a result of these metals accumulating on the surface of the Ti-Al alloys, which simultaneously enhanced the hydrogen evolution efficiency and inhibited metal dissolution [99].

Overall, light metallic systems such as Ti and Al alloys are essential in the biomedical and aeronautical industries. Additively-manufactured components require controlled processing in order to avoid non-equilibrium phase precipitation and prevent the decreasing of corrosion resistance. Further research is required to understand the influence of some AM processes parameters on the corrosion resistance of Al and Ti components, as well as their improvement in comparison to conventional processed parts.

## 5. Duplex Stainless Steels and Additive Manufacturing

DSS are widely investigated due to both their mechanical and corrosive properties, although only 1.558 articles were published for the last five years considering their processing through AM techniques and their influence on corrosion resistance. Therefore, in this section, the main features of AM fabrication and problems related to corrosion resistance of duplex stainless steels (DSS) are presented, these being the most widely fabricated through AM processes among stainless steel types.

Duplex stainless steels have extensive applications as structural materials in many industries and different environments, as these steels present both high mechanical and corrosion resistance properties provided by their dual-phase microstructure (ferrite δ and austenite γ) [100]. Some of their applications involve chemical, oil, gas, food, and marine industries, where their good performance and attractive economical cost can substitute alternative materials such as nickel-based alloys. These steels may present precipitation of undesired intermetallic phases, carbides, and nitrides at different temperatures. Particularly, from 600 °C to 1000 °C, the sigma phase (σ), chi-phase (χ), carbides (M_23_C_6_, M_7_C_3_), or nitrides (CrN, Cr_2_N) can precipitate and deteriorate their mechanical properties as well as decrease their corrosion resistance [101,102,103]. Moreover, the intermetallic phases present higher content in Fe, Cr, and Mo and, thus, can be the main factor responsible for the decrease in toughness and pitting corrosion. Due to the ferrite-formation elements (Fr, Cr, and Mo), these secondary phases are considered to precipitate from the ferrite phase and, in some cases, can lead to its total consumption.

To show the complexity of the microstructure that can appear when processing DSS by AM, it is worth noting that there is some controversy around the transformation from delta ferrite during heat treatment after the conventional casting processing which has been just recently solved [104]. The usual microstructure observed in a typical wrought microstructure is composed of ferrite (47 ± 4)% and austenite (53 ± 4)%. Hot-rolling materials provided numerous potential void nucleation sites, which accelerated the propagation of cracks along the ferrite/austenite interface and caused serious cracking [105]. Meanwhile, the weld material has a typical microstructure of ferrite (42.1 ± 3.0)% and Widmanstätten austenite (57.9 ± 3.0)% as reported by Tavares, et al. [106], with massive plates of σ phase precipitated in the ferrite phase.

Because of the attractive properties of the ferritic-austenitic microstructure, and, therefore, the wide range of applications that DSS can perform, industry is increasingly processing DSS through AM techniques. Such processing not only allows for obtaining different shapes and dense materials, but can also be an alternative process to the conventional techniques. However, some of the main problems with these processing techniques are related to the precipitation of undesired secondary phases and the balance control of the two main phases in order to maintain desirable mechanical properties and, more importantly, the corrosion resistance properties, because of the severe conditions in which they are used. In order to simulate these conditions, most of the corrosion studies used (mainly polarization tests) in the literature are carried out under similar conditions; for instance, 3.5 wt% NaCl solutions at room temperature. Thus, in the following section, the corrosion results are presented for some typical grades of duplex stainless steels.

### 5.1. Main AM Processes and Associated Problems

Additively manufactured steels can be more susceptible to corrosion due to several defects that may occur during processing. Not only the presence of intermetallic phases but also microstructural defects after the processes such as porosity because of incomplete fusion between successive molten layers or localized defects can be of importance when considering the decrease in corrosion resistance. However, in some cases, for example during SLM processing, porosity is highly improved [107,108]. A refined grained microstructure can be achieved and, thus, pitting corrosion resistance is increased. In addition, a higher number of grain boundaries promotes the diffusion of elements which could form stable passive films. Research has shown that different AM stainless steels perform better with regards to corrosion resistance than the same wrought stainless steels. Moreover, as seen above, most of the AM processes for stainless steels require controlled cooling rates and heat treatments in order to obtain an optimal microstructure and balanced austenite-ferrite ratio to maintain the desirable corrosion resistance properties. The general behavior and pitting corrosion resistance of some common duplex stainless-steel grades after being AM processed are discussed in the following.

#### 5.1.1. Wire Arc Additive Manufacturing Processes

One of the most-used AM processes with DSS is the wire arc additive manufacturing process (WAAM), which is a direct energy deposition process, where filler wire and arc are the heating source. This process is cheaper and more readily implemented in industries, as well as enabling higher productivity. However, as mentioned above, one of the main problems associated with its performance is the maintaining of an optimal dual ferritic-austenitic microstructure. Because of the continuous reheating process by forming consecutive layers, it is of great importance to control the heat input, the interpass temperatures, and the high cooling rates in order to avoid undesired intermetallic phases and to maintain the balance between ferrite and austenite.

**Figure 5:**
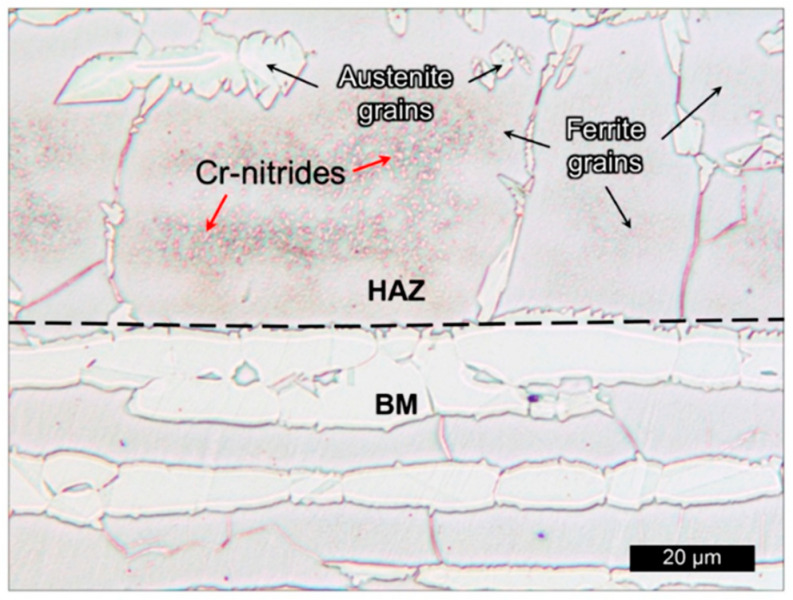
Chromium nitrides in HAZ in a SDSS 2507 superduplex steel (SDSS) as support plate (BM) and SDSS wire after WAAM process [111].

There are several studies describing the material properties and process effects such as temperature on phase precipitation, performance, and phase balance in DSS [41,109,110,111,112,113] after WAAM. For instance, Eriksson et al. [109] studied the effect of heat input on the mechanical properties of superduplex stainless steel UNS S32507, considering that harmful secondary phases could be formed during the process. They have shown that the base metal consisted of 48 vol% ferrite and 52 vol% austenite; however, during welding, the microstructure transforms to ferrite. Moreover, the precipitation of austenite from ferrite during cooling depends exclusively on the cool rate. The heat-affected zone showed a higher content of ferrite than the welded zone, between 49 vol% and 56 vol%, depending on the heat input. In addition, the highest content of ferrite was found in the top layer, as there was no subsequent layer, thus no reheating. By taking care of the heat deposition and of the cooling rate, a promising microstructure without sigma secondary phase precipitation and enhanced mechanical properties can be achieved. Later, Lervåg et al. [111] showed the precipitation of chromium nitrides, CrN, mainly in the HAZ rather than in the base material (BM) in the same superduplex stainless steel UNS S32507, as seen in Figure 5. Such precipitation is found in the ferrite phase after the rapid cooling from high temperatures, due to the supersaturation of nitrogen in the ferrite. Thus, chromium nitrides were not found in the built walls after WAAM, due to the lower content of ferrite phase. The formed layers of the material were obtained through the Cold Metal Transfer (CMT) process-based WAAM technique under different heat inputs in the range of 0.40–0.87 kJ/mm and the absence of intermetallic phases at low heat input was reported. The lower heat input is due to the reciprocating motions of the filler wire and the short-circuiting mode of the material, which produce reduced sputter in comparison to the conventional arc mode [101].

Hejripour et al. [112] demonstrated that a controlled cooling rate promotes austenite formation in ferrite matrix and prevents the formation of intermetallic phases during the process of building two different parts of 2209 DSS using the WAAM process. However, due to the complex thermal cycles and cooling rates, the ferrite content after the process showed a lower quantity in comparison to the same material processed by the conventional technique. Due to the required high cooling rate in the first layer (shorter distance between substrate and layer), a finer microstructure was obtained. The sigma phase and other deleterious intermetallic phases were not noticed in the microstructure and the ferrite content in the WAAM parts was lower than in the base metal produced by conventional methods.

Posch et al. [114] have shown the successful application of the CMT-WAAM (Cold Metal Transfer-WAAM) process to build blade-shaped geometry by a robot layer-by-layer using 2209 duplex stainless steel filler wire. No porosity, and non-harmful phases were detected in the transition microstructure of two scams, and a δ-ferrite content of 26 vol%–29 vol% was found by EBSD analyses. Therefore, the microstructure of the additively manufactured DSS is comparable to conventional GMAW DSS weld metals. The EBSD phase mapped the ferrite (δ)/austenite (γ) microstructure of the material after the process without any further heat treatment.

The flux-cored WAAM (FCWAAM) process is another technique to produce DSS weld metal, consisting of flux-cored wire instead of solid wires. Hence, there is more adjustability in the wire composition in order to better control the obtained microstructure of the fabricated material. Zhang et al. [115] have also found an excessive austenite content (around 74% average ratio) in a DSS component, using commercial 2009 DSS welding wire as filling material in the Flux-cored WAAM process. Although the mechanical properties of the obtained component were desirable, there was a difference in properties in the bottom and top layers in the horizontal direction. Therefore, efforts must be made to promote a balanced ferrite (δ)/austenite (γ) microstructure. For instance, authors have made heat treatments afterwards at 1250 °C and 1300 °C in order to achieve an approximately equal ratio of both austenite and ferrite phases, obtaining promising pitting corrosion resistance which is comparable to a hot-rolled 2205 DSS plate. Another alternative that allowed researches to modify the austenite ratio was developing specific wires for the WAAM process of DSS by altering the composition of the flux in the flux-cored wire. For example, Cr content was higher and Ni content was lower in the new FCWAAM DSS components in comparison to the commercial welding wire.

Later, Zhang et al. [116] showed, in another investigation, the satisfying pitting corrosion resistance of heat-treated WAAM DSS components, comparable to as-built WAAM DSS and hot-rolled 2205 DSS. The recommended post-AM heat treatment temperature for WAAM DSS was 1300 °C. In addition, the corrosion behavior of the as-built components was worse than the hot-rolled 2205 DSS, because of the different austenite ratio and chromium nitrides (Cr2N) and a chemically different composition of austenite, the as-known secondary austenite (γ2) were detected. The lack of chromium nitrides after WAAM DSS were heat treated at 1250 °C and 1300° showed desirable pitting corrosion resistance.

Tavares et al. [117] reported results on the corrosion behavior of superduplex stainless steel 2705. Similar to a 2205 duplex stainless steel, a balanced microstructure was crucial for preventing pitting decrease. For instance, PREN over 40 was verified by Rajesh et al. [118] for a SDSS 2594 after WAAM process, showing the similar behavior of three different parts of the material, the base metal, middle part, and top layer, and their better pitting corrosion resistance in comparison to the same composition wrought alloy. The obtained microstructure showed no traces of intermetallic phases, which was reflected in the excellent pitting corrosion properties. Moreover, the similar corrosion behavior of three different parts in the wall layers showed a homogeneous microstructure after the process. However, some transients in current, indicating the occurrence of repassivating pitting events, were observed. Such transient pitting coincides with the middle layer, which is expected to have the higher load of defects. Defects such as LOF, porosity, or residual stresses may influence the corrosion resistance of the material; thus, the optimization of the layer deposition in addition to the control of the intermetallic phase formation is of crucial importance.

Overall, it could be summarized that WAAM processes are based on a deposition of subsequent layers and, thus, continuous heating occurs during the process, requiring extra high cooling rates because of the dissipation of the heat along and perpendicularly to the deposition direction [118,119]. On the contrary, when slower cooling rates are performed, there is enough time for the transformation of ferrite to austenite, leading to an excessive austenite ratio in the final layers. Obtaining a balanced austenite/ferrite microstructure is still a challenge in comparison to other processes. There are alternative techniques such as CMT-WAAM processes or the flux-cored wire used of FC-WAAM and, due to the lower heat input or flux-cored wires, the excessive austenite ratio could be modified. Moreover, post-heat treatments are mostly required.

#### 5.1.2. Selective Laser Melting Processes

Selective laser melting (SLM) is another of the powder bed fusion techniques that are widely used as AM processing for duplex stainless steels. After SLM processing, the as-built microstructure of DSS is almost ferritic. Consequently, solution annealing is required afterwards for achieving a balanced ferrite/austenite microstructure [120,121]. On the other hand, due to temperature changes, resulting from the fast-thermal heating and cooling cycles, a high number of residual stresses appear in the as-built microstructure during and after SLM processing. Such residual stresses can be removed during the post-annealing treatment or by reducing their presence by decreasing the temperature gradient at the initial point of the process by preheating the building platform [122].

Several authors have shown microstructural features that manifest the importance of the heat-annealing treatment after applying SLM. Papula et al. [122] determined that, to obtain a ferrite-austenite microstructure, 2205 DSS had to be solution-annealed from 950 °C to 1100 °C after the SLM process. In addition, the as-built SLM-processed material presented an almost fully ferritic microstructure (99%) and, after the corresponding heat treatment, 40–46% of austenitic content was reached. The as-built SLM-processed material was mainly formed of ferrite phase, whereas heat-treated samples for different times showed an austenite phase increment up to 46%.

Shang et al. [123] observed, using EBSD, that secondary phases, such as sigma, were precipitated after SLM processing on 2707 HDSS. However, after the same processing and additional solution annealing treatments, at different temperatures, it gave place to a balanced ferrite-austenite microstructure. Characterization after solution annealing showed that the temperature must be carefully selected, as the sigma phase was still detected and mostly austenite phase was present in the microstructure. The corrosion behavior was evaluated and a particular ratio of α to γ showed better corrosion resistance. Therefore, phase arrangement is responsible for the higher pitting resistance compared to the other samples wherein which the precipitation of intermetallic sigma phase has occurred. Thus, the content of ferrite phase/vol.% was varied. σ phase is mainly found at α/α, α/γ, and γ/γ, and it may have led to depleted Cr areas at such phase boundaries, which further resulted in decreased pitting resistance. On the other hand, passive films were also investigated and showed better stability within the increase in grain boundaries due to the grain refinement. Thus, an improved passive film is formed due to the improved chromium distribution [124].

Davidson et al. [125] reported a high content of ferrite phase in a 2705 SDSS after the SLM process with some chromium nitrides. However, after a heat treatment, the ratio changed again. The high cooling rate after the process mainly restricts the austenite phase nucleation and an almost fully ferrite microstructure is observed in the as-built material. After a suitable heat treatment, a balanced austenite-ferrite microstructure is obtained, as was also seen by Hengsback et al. [126] for 1803 DSS material after the SLM process.

Table 4 shows a summary of different duplex stainless steel grades processed by WAAM and SLM processes, the post-AM heat treatments applied, and the pitting corrosion potential.

In conclusion, additive manufacturing processes are a promising alternative method to fabricate duplex stainless steels, especially when complex and irregular shapes are required in addition to the density of the materials, in comparison to the conventional techniques. However, high cooling rates and control during the processes are of extreme importance in order to avoid the formation of deleterious secondary phases that can affect mechanical and corrosion resistance properties.

On the other hand, it is crucial to maintain the balanced ferrite/austenite microstructure. Hence, most of the reviewed investigations have tried to modify the ratio of austenite by combining WAAM with other process, for instance, using cold wire feed [119] or using heat treatments after the AM process [127,128]. The high cooling rates and, most of the time, additional treatments are crucial; for instance, post-AM heat treatments are required to balance the austenite/ferrite ratio and maintain a desirable pitting corrosion resistance of duplex stainless steels.

AM processes such as SLM or EBM present some undesirable effects or limitations. The latest research is looking further into WAAM processes and their relationship to corrosion resistance [129], as well as new and recently-developed μ-plasma arc additive manufacturing process, which are quite promising [130].

Finally, AM is still an emergent manufacturing concept with many parameters in consideration to obtain the “perfect” component. However, attending the latest 2022 trends, there is a new concept in discussion related to the corrosion proofing of AM alloy parts. For instance, the low-temperature infusion of interstitial solute, exemplified for a high-entropy alloy (Alloy 22) to simulate thermochemical treatment, has been discussed by Illing et al. [131]. The post-processing of these parts is carried out through a newly developed process of LTNC-SRP (low-temperature nitrocarburization by solid-reagent pyrolysis). Future research should focus on the definition of proper corrosion tests to characterize the strong relationship between complex AM microstructures and corrosion resistance with accuracy. It should take into consideration the role of each defect, since each defect could create local electrochemical conditions leading to local corrosion cells. In respect, there should be a normal standard for these types of materials as they are processed by AM. In order to assess and develop apropiate testing for qualification purposes, it is critical to understand the long-term performance of AM materials in different corosion environments and correlate them to the short-term laboratory studies, which is also discussed in reference [7].

## 6. Conclusions

Additive manufacturing is an attractive alternative manufacturing technique for metallic components. AM allows for the fabrication of dense materials with complex shapes and comparable properties to the same conventionally manufactured materials. In this review, different AM processes, the most commonly occurring defects, and the corrosion resistance of light metals, duplex stainless steels, and other metallic materials obtained by different AM processes have been analyzed.

It has been reported by many researchers that light metals such as Ti and Al alloys may present some problems during the AM process, and are mainly related to the processing temperature that drives the formation of different intermetallic phases or oxides that could act as defect nucleation site with their impact on corrosion behavior. It is necessary to point out that more studies on the corrosion behavior of AM, in particular SLM-produced materials, are needed to understand the corrosion resistance of the AM-produced parts related to AM biomedical Ti alloys.

The results of adding Pt group metals to other metallic systems, with the improvement in their corrosion resistance, gives some interesting expectations to enhance the corrosion properties of other composites, even in switching to the AM technique.

Phase precipitation is one of the main problems related to the AM techniques for duplex stainless steels. Moreover, it is the maintenance of the dual phase microstructure that is of crucial importance in order to provide comparable mechanical and corrosion resistance properties to the conventionally fabricated materials. It has been found in the literature that AM could be a successful and powerful process with regards to pitting corrosion resistance properties, whenever austenite/ferrite ratio is maintained. With most of the AM processes, the as-built DSS components showed an almost fully ferrite microstructure and, thus, post-AM heat treatments are required to balance the austenite ratio.

Corrosion testing of AM components must take into account the high degree of anisotropy of all kinds of defects, which may appear in such objects, depending on the kind of process. When planning corrosion tests, the correct grinding and polishing procedures (allowing no modification of morphology or size of the defects, especially for surface porosity), the surface after post-processing (if applicable), the bulk material—tested surface perpendicular to the build direction (depth is relevant), and bulk material—tested surface parallel to the build direction must be considered. Not all of these test variables will be relevant for the corrosion susceptibility under service conditions; however, they may yield valuable information on the kind and distribution of defects on and in the AM material.

Finally, it must be pointed out that a real effort should be undergone to further investigate the corrosion behavior of metal additive manufacturing alloys, since there is not much literature that has studied it. For the last seven years, from 2015 to 2022, there have been more than 20,000 published studies that consider additively manufactured metallic systems. The majority of these studies are related to mechanical properties, mainly fatigue behavior and dimensional issues of the components. The existence of alternative methods to analyze corrosion behavior as LRP could be the beginning of understanding the interrelation between manufacture variables, as well as the impacts of microstructure and corrosion resistance. Carrying out the right actions to improve corrosion behavior and preventing its decrease is crucial to avoid undesirable situations.

## Figures and Tables

**Table 1:** Main advantages and disadvantages of DED and PBF AM techniques.

Main Process		Advantages	Disadvantages	References
DED	LMD	Less micro-cracking Improved thermal control	Require post-processing Low process rates	[40]
	WAAM	Good structural integrity High deposition rate	Lower accuracy Different microstructure obtained	[24,40,41]
PBF	L-PBF(SLM)	High heat and process speed No support structure required	Surface roughness Powder particle size	[42,43]
	EBM	High process rates Good accuracy Fully dense parts	High surface roughness Requires high-quality powder	[42,44,45]

**Table 2:** Major reported defects of various metallic systems processed by AM.

Material	Process	Type Defect	Reported by
Inconel	LMD	Gas Porosity	[54,64,70]
Stainless Steel	SLW, SLM	LOF, porosity	[23,57,59,74]
CoCrMo alloy	PBFAM	lack-of-fusion flaws	[57]
Ti alloys	SLM	Flaws	[56,58]
Al alloys	WAAM	Hydrogen porosity, oxides	[63,69]
Fe superalloys	WAAM, LPBF	Brittle phases, microcracks	[75,76]

**Table 3:** Associated problems to MAM and their solutions.

Problem	Cause	Solution
Refrigeration	Materials are not able to withstand the heat generated during AM.	Add external part that cools the equipment as an electronic chassis [77].
Material cost	Lightness of the materials makes it difficult to measure them accurately until there are not enough layers.	Creation of different gadgets to resolve this issue [78].
Abruply deposition	Control of deposition is not accurate due to the usually lower melting point of light metals	Add intermediate piece on which the molten material accumulates before being deposited or a first laser that pre-fuses the material [79].

**Table 4:** Different DSS types processed by AM and post-AM heat treatments revealing critical issues related to the corrosion behaviour.

Material	Main Process	Solution Annealing [°C]	Qualitative Corrosion Aspects	Reference
2205 DSS	WAAM	1250–1350	Balanced ratio ferrite-austenite; improved pitting corrosion resistance after 1300 °C heat treatment	[116]
2594 SDSS	WAAM	-	Stable micropits in the middle part without decreasing corrosion resistance	[118]
2205 DSS	SLM	950–1100	Improved pitting resistance after heat treatments	[122]
2707 HDSS	SLM	1050–1200	Balanced ratio ferrite-austenite; higher pitting corrosion resistance after solution annealing	[123]

## Data Availability

Data sharing not applicable.

## Glossary

Abbreviation	Item
AM	Additive Manufacturing
BM	Base Metal
CMT	Cold Metal Transfer
CNC	Computer Numerical Control
CT	Computed Tomography
DED	Direct Energy Deposition
DLD	Direct Laser Deposition
DSS	Duplex Stainless Steels
EBM	Electron Beam Melting
EBSD	Electron Backscatter Diffraction
FC	Flux-Cored
FGM	Functionally Graded Materials
GMAW	Gas Metal Arc Welding
GP	Gas Porosity
HAZ	Heat-Affected Zone
HDSS	Hyperduplex Stainless Steels
HIP	Hot Isostatic Pressing
LMD	Laser Metal Deposition
LOF	Lack-of-Fusion
LTNC-SRP	Low temperature nitrocarburization by solidreagent pyrolisis
MAM	Metal additive manufacturing
MPB	Melt Pool Border
MPZ	Melt Pool Zone
NAB	Nickel-Aluminium-Bronze alloys
PBF	Powder Bed Fusion
PBFAM	Powder bed fusion additive manufacturing
SDSS	Superduplex Stainless Steels
SLM	Selective Laser Melting
SLS	Selective Laser Sintering
VSCE	Electrode potential measured versus Saturated Calomel Electrode
WAAM	Wire Arc Additive Manufacturing

## References

[1] Culmone C., Smit G., Breedveld P. (2019). Additive manufacturing of medical instruments: A state-of-the-art review. Addit. Manuf..

[2] Zhang Q., Guan Y. (2023). Application of metal additive manufacturing in oral dentistry. Curr. Opin. Biomed. Eng..

[3] Seiti M., Ginestra P. (2023). Additive Manufacturing for orthopedic applications: Case study on market impact. Procedia Comput. Sci..

[4] Artaza T., Suárez A., Veiga F., Braceras I., Tabernero I., Larrañaga O., Lamikiz A. (2020). Wire arc additive manufacturing Ti6Al4V aeronautical parts using plasma arc welding: Analysis of heat-treatment processes in different atmospheres. J. Mater. Res. Technol..

[5] Madhavadas V., Srivastava D., Chadha U., Raj S.A., Sultan M.T.H., Shahar F.S., Shah A.U.M. (2022). A review on metal additive manufacturing for intricately shaped aerospace components. CIRP J. Manuf. Sci. Technol..

[6] Monteiro H., Carmona-Aparicio G., Lei I., Despeisse M. (2022). Energy and material efficiency strategies enabled by metal additive manufacturing—A review for the aeronautic and aerospace sectors. Energy Rep..

[7] Schindelholz E.J., Melia M.A., Rodelas J.M. (2021). Corrosion of Additively Manufactured Stainless Steels—Process, Structure, Performance: A Review. Corrosion.

[8] Sander G., Tan J., Balan P., Gharbi O., Feenstra D., Singer L., Thomas S., Kelly R., Scully J., Birbilis N. (2018). Corrosion of Additively Manufactured Alloys: A Review. Corrosion.

[9] Praveena B.A., Lokesh N., Buradi A., Santhosh N., Praveena B.L., Vignesh R. (2021). A comprehensive review of emerging additive manufacturing (3D printing technology): Methods, materials, applications, challenges, trends and future potential. Mater. Today Proc..

[10] Orzolek S.M., Semple J.K., Fisher C.R. (2022). Influence of processing on the microstructure of nickel aluminum bronze (NAB). Addit. Manuf..

[11] (2015). Additive Manufacturing-General Principles-Terminology.

[12] Kumar S.A., Prasad R. (2021). Basic principles of additive manufacturing: Different additive manufacturing technologies. Additive Manufacturing.

[13] Frazier W.E. (2014). Metal Additive Manufacturing: A Review. J. Mater. Eng. Perform..

[14] Faludi J., Bayley C., Bhogal S., Iribarne M. (2015). Comparing environmental impacts of additive manufacturing vs traditional machining via life-cycle assessment. Rapid Prototyp. J..

[15] Javaid M., Ariz A., Tasneem I., Bharti D., Vaish A., Haleem A. (2021). Is additive manufacturing of patient-specific implant is beneficial for orthopedics. Apollo Med..

[16] Pereira T., Kennedy J.V., Potgieter J. (2019). A comparison of traditional manufacturing vs additive manufacturing, the best method for the job. Procedia Manuf..

[17] Fathi P., Mohammadi M., Duan X., Nasiri A.M. (2019). A comparative study on corrosion and microstructure of direct metal laser sintered AlSi10Mg_200C and die cast A360.1 aluminum. J. Mater. Process. Technol..

[18] Rafi H.K., Karthik N.V., Gong H., Starr T.L., Stucker B.E. (2013). Microstructures and Mechanical Properties of Ti6Al4V Parts Fabricated by Selective Laser Melting and Electron Beam Melting. J. Mater. Eng. Perform..

[19] Zhai Y., Galarraga H., Lados D.A. (2016). Microstructure, static properties, and fatigue crack growth mechanisms in Ti-6Al-4V fabricated by additive manufacturing: LENS and EBM. Eng. Fail. Anal..

[20] Revilla R.I., Verkens D., Rubben T., De Graeve I. (2020). Corrosion and Corrosion Protection of Additively Manufactured Aluminium Alloys—A Critical Review. Materials.

[21] Dawes J., Bowerman R., Trepleton R. (2015). Introduction to the Additive Manufacturing Powder Metallurgy Supply Chain. Johns. Matthey Technol. Rev..

[22] Martin J.H., Yahata B.D., Hundley J.M., Mayer J.A., Schaedler T.A., Pollock T.M. (2017). 3D printing of high-strength aluminium alloys. Nature.

[23] Sander G., Thomas S., Cruz V., Jurg M., Birbilis N., Gao X., Brameld M., Hutchinson C.R. (2017). On The Corrosion and Metastable Pitting Characteristics of 316L Stainless Steel Produced by Selective Laser Melting. J. Electrochem. Soc..

[24] Sun G., Shen X., Wang Z., Zhan M., Yao S., Zhou R., Ni Z. (2018). Laser metal deposition as repair technology for 316L stainless steel: Influence of feeding powder compositions on microstructure and mechanical properties. Opt. Laser Technol..

[25] Trelewicz J.R., Halada G.P., Donaldson O.K., Manogharan G. (2016). Microstructure and Corrosion Resistance of Laser Additively Manufactured 316L Stainless Steel. JOM.

[26] Zhang X., Lv Y., Tan S., Dong Z., Zhou X. (2021). Microstructure and corrosion behaviour of wire arc additive manufactured AA2024 alloy thin wall structure. Corros. Sci..

[27] McAndrew A.R., Rosales M.A., Colegrove P.A., Hönnige J.R., Ho A., Fayolle R., Eyitayo K., Stan I., Sukrongpang P., Crochemore A. (2018). Interpass rolling of Ti-6Al-4V wire + arc additively manufactured features for microstructural refinement. Addit. Manuf..

[28] Bhavar V., Kattire P., Patil V., Khot S., Gujar K., Singh R. (2018). A review on powder bed fusion technology of metal additive manufacturing. Additive Manufacturing Handbook.

[29] Kong D., Dong C., Ni X., Li X. (2019). Corrosion of metallic materials fabricated by selective laser melting. npj Mater. Degrad..

[30] Otero E., Pardo A., Utrilla V., Saenz E., Álvarez J. (1998). Corrosion behaviour of aisi 304l and 316l stainless steels prepared by powder metallurgy in the presence of sulphuric and phosphoric acid. Corros. Sci..

[31] Maximenko A.L., Olevsky E.A. (2018). Pore filling during selective laser melting—Assisted additive manufacturing of composites. Scr. Mater..

[32] Housholder R.F. (1979). Molding Process. U.S. Patent.

[33] Beaman J.J., Deckard C.R. (1989). Selective Laser Sintering with Assisted Powder Handling. U.S. Patent.

[34] Al-Shebeeb O.A. (2021). An Investigation of the Metal Additive Manufacturing Issues and Perspective for Solutions Approach. Concepts, Applications and Emerging Opportunities in Industrial Engineering.

[35] Ma F., Zhang H., Hon K., Gong Q. (2018). An optimization approach of selective laser sintering considering energy consumption and material cost. J. Clean. Prod..

[36] Vayre B., Vignat F., Villeneuve F. (2012). Metallic additive manufacturing: State-of-the-art review and prospects. Mech. Ind..

[37] Richardson B.S., Lind R.F., Lloyd P.D., Noakes M.W., Love L.J., Post B.K. (2018). The design of an additive manufactured dual arm manipulator system. Addit. Manuf..

[38] Murr L. (2015). Metallurgy of additive manufacturing: Examples from electron beam melting. Addit. Manuf..

[39] Zafer Y.E., Goel S., Ganvir A., Jansson A., Joshi S. (2020). Encapsulation of Electron Beam Melting Produced Alloy 718 to Reduce Surface Connected Defects by Hot Isostatic Pressing. Materials.

[40] Najmon J.C., Raeisi S., Tovar A. (2019). Review of additive manufacturing technologies and applications in the aerospace industry. Additive Manufacturing for the Aerospace Industry.

[41] Jin W., Zhang C., Jin S., Tian Y., Wellmann D., Liu W. (2020). Wire Arc Additive Manufacturing of Stainless Steels: A Review. Appl. Sci..

[42] Gao W., Zhang Y., Ramanujan D., Ramani K., Chen Y., Williams C.B., Wang C.C.L., Shin Y.C., Zhang S., Zavattieri P.D. (2015). The status, challenges, and future of additive manufacturing in engineering. Comput. Aided Des..

[43] Coemert S., Traeger M.F., Graf E.C., Lueth T.C. (2017). Suitability Evaluation of various Manufacturing Technologies for the Development of Surgical Snake-like Manipulators from Metals Based on Flexure Hinges. Procedia CIRP.

[44] Bartlett J.L., Li X. (2019). An overview of residual stresses in metal powder bed fusion. Addit. Manuf..

[45] Jabnoun N. (2002). Control processes for total quality management and quality assurance. Work. Study.

[46] Webster S., Lin H., Iii F.M.C., Ehmann K., Cao J. (2021). Physical mechanisms in hybrid additive manufacturing: A process design framework. J. Mater. Process. Technol..

[47] Wu B., Pan Z., Ding D., Cuiuri D., Li H., Xu J., Norrish J. (2018). A review of the wire arc additive manufacturing of metals: Properties, defects and quality improvement. J. Manuf. Process..

[48] Kim F.H., Moylan S.P. (2018). Literature Review of Metal Additive Manufacturing Defects.

[49] Busachi A., Erkoyuncu J., Colegrove P., Martina F., Ding J. (2015). Designing a WAAM Based Manufacturing System for Defence Applications. Procedia CIRP.

[50] Zhang B., Li Y., Bai Q. (2017). Defect Formation Mechanisms in Selective Laser Melting: A Review. Chin. J. Mech. Eng..

[51] Xu Z., Wang Q., Wang X., Tan C., Guo M., Gao P. (2020). High cycle fatigue performance of AlSi10mg alloy produced by selective laser melting. Mech. Mater..

[52] Siddique S., Imran M., Rauer M., Kaloudis M., Wycisk E., Emmelmann C., Walther F. (2015). Computed tomography for characterization of fatigue performance of selective laser melted parts. Mater. Des..

[53] Rollett A. Finding Keyholes in Metal Additive Manufacturing|2020-03-11|Industrial Heating. https://www.industrialheating.com/articles/95529-finding-keyholes-in-metal-additive-manufacturing.

[54] Khairallah S.A., Martin A.A., Lee J.R.I., Guss G., Calta N.P., Hammons J.A., Nielsen M.H., Chaput K., Schwalbach E., Shah M.N. (2020). Controlling interdependent meso-nanosecond dynamics and defect generation in metal 3D printing. Science.

[55] Collins P.C., Bond L.J., Taheri H., Bigelow T.A., Shoaib M.R.B.M., Koester L.W. (2017). Powder-based additive manufacturing—A review of types of defects, generation mechanisms, detection, property evaluation and metrology. Int. J. Addit. Subtractive Mater. Manuf..

[56] Snow Z., Diehl B., Reutzel E.W., Nassar A. (2021). Toward in-situ flaw detection in laser powder bed fusion additive manufacturing through layerwise imagery and machine learning. J. Manuf. Syst..

[57] Nassar A.R., Gundermann M.A., Reutzel E.W., Guerrier P., Krane M.H., Weldon M.J. (2019). Formation processes for large ejecta and interactions with melt pool formation in powder bed fusion additive manufacturing. Sci. Rep..

[58] Monzón Vivas A. Thickness and Orientation Dependent Surface Roughness and Internal Defect Characterization of SLM Ti-6Al-4V. https://riunet.upv.es/handle/10251/163329.

[59] Blinn B., Klein M., Gläßner C., Smaga M., Aurich J.C., Beck T. (2018). An Investigation of the Microstructure and Fatigue Behavior of Additively Manufactured AISI 316L Stainless Steel with Regard to the Influence of Heat Treatment. Metals.

[60] Roehling T.T., Wu S.S., Khairallah S.A., Roehling J.D., Soezeri S.S., Crumb M.F., Matthews M.J. (2017). Modulating laser intensity profile ellipticity for microstructural control during metal additive manufacturing. Acta Mater..

[61] Gu J., Ding J., Williams S.W., Gu H., Ma P., Zhai Y. (2016). The effect of inter-layer cold working and post-deposition heat treatment on porosity in additively manufactured aluminum alloys. J. Mater. Process. Technol..

[62] Derekar K., Lawrence J., Melton J., Addison A., Zhang X., Xu L. (2019). Influence of interpass temperature on wire arc additive manufacturing (WAAM) of aluminium alloy components. Proceedings of the IIW 2018—International Conference on Advanced Welding and Smart Fabrication Technologies.

[63] Hauser T., Reisch R.T., Breese P.P., Lutz B.S., Pantano M., Nalam Y., Bela K., Kamps T., Volpp J., Kaplan A.F. (2021). Porosity in wire arc additive manufacturing of aluminium alloys. Addit. Manuf..

[64] Ibrahim H., Jahadakbar A., Dehghan A., Moghaddam N.S., Amerinatanzi A., Elahinia M. (2018). In Vitro Corrosion Assessment of Additively Manufactured Porous NiTi Structures for Bone Fixation Applications. Metals.

[65] Qiu X. (2021). Microstructure and corrosion properties of Al2CrFeCo CuNiTi high entropy alloys prepared by additive manufacturing. J. Alloy. Compd..

[66] Javadi Y., MacLeod C.N., Pierce S.G., Gachagan A., Lines D., Mineo C., Ding J., Williams S., Vasilev M., Mohseni E. (2019). Ultrasonic phased array inspection of a Wire+ Arc Additive Manufactured (WAAM) sample with intentionally embedded defects. Addit. Manuf..

[67] Ye D., Hong G.S., Zhang Y., Zhu K., Fuh J.Y.H. (2018). Defect detection in selective laser melting technology by acoustic signals with deep belief networks. Int. J. Adv. Manuf. Technol..

[68] Romano S., Abel A., Gumpinger J., Brandão A., Beretta S. (2019). Quality control of AlSi10Mg produced by SLM: Metallography versus CT scans for critical defect size assessment. Addit. Manuf..

[69] Nourian-Avval A., Fatemi A. (2020). Characterization and Analysis of Porosities in High Pressure Die Cast Aluminum by Using Metallography, X-Ray Radiography, and Micro-Computed Tomography. Materials.

[70] Ng G.K.L., Jarfors A.E.W., Bi G., Zheng H.Y. (2009). Porosity formation and gas bubble retention in laser metal deposition. Appl. Phys. A.

[71] Kang Y.-J., Yang S., Kim Y.-K., AlMangour B., Lee K.-A. (2019). Effect of post-treatment on the microstructure and high-temperature oxidation behaviour of additively manufactured inconel 718 alloy. Corros. Sci..

[72] Chandrasekaran S., Hari S., Amirthalingam M. (2020). Wire arc additive manufacturing of functionally graded material for marine risers. Mater. Sci. Eng. A.

[73] Bobbio L.D., Otis R.A., Borgonia J.P., Dillon R.P., Shapiro A.A., Liu Z.-K., Beese A.M. (2017). Additive manufacturing of a functionally graded material from Ti-6Al-4V to Invar: Experimental characterization and thermodynamic calculations. Acta Mater..

[74] Shang Y., Yuan Y., Li D., Li Y., Chen J. (2017). Effects of scanning speed on in vitro biocompatibility of 316L stainless steel parts elaborated by selective laser melting. Int. J. Adv. Manuf. Technol..

[75] Han Q., Gu Y., Soe S., Lacan F., Setchi R. (2019). Effect of hot cracking on the mechanical properties of Hastelloy X superalloy fabricated by laser powder bed fusion additive manufacturing. Opt. Laser Technol..

[76] Qiu Z., Wu B., Zhu H., Wang Z., Hellier A., Ma Y., Li H., Muransky O., Wexler D. (2020). Microstructure and mechanical properties of wire arc additively manufactured Hastelloy C276 alloy. Mater. Des..

[77] Kim J.H., Anderson J., Hoden B. (2021). Electronics Chassis with Oscillating Heat Pipe (OHP). U.S. Patent.

[78] US11097487Patents|PatentGuru. https://www.patentguru.com/search?q=US11097487.

[79] US11097350Patents|PatentGuru. https://www.patentguru.com/search?q=US11097350.

[80] Monteiro W.A. (2014). Light Metal Alloys Applications.

[81] Aboulkhair N.T., Simonelli M., Parry L., Ashcroft I., Tuck C., Hague R. (2019). 3D printing of Aluminium alloys: Additive Manufacturing of Aluminium alloys using selective laser melting. Prog. Mater. Sci..

[82] Maamoun A.H., Xue Y.F., Elbestawi M.A., Veldhuis S.C. (2018). The Effect of Selective Laser Melting Process Parameters on the Microstructure and Mechanical Properties of Al6061 and AlSi10Mg Alloys. Materials.

[83] Leon A., Aghion E. (2017). Effect of surface roughness on corrosion fatigue performance of AlSi10Mg alloy produced by Selective Laser Melting (SLM). Mater. Charact..

[84] Nezhadfar P., Thompson S., Saharan A., Phan N., Shamsaei N. (2021). Structural integrity of additively manufactured aluminum alloys: Effects of build orientation on microstructure, porosity, and fatigue behavior. Addit. Manuf..

[85] Amanov A. (2020). Effect of local treatment temperature of ultrasonic nanocrystalline surface modification on tribological behavior and corrosion resistance of stainless steel 316L produced by selective laser melting. Surf. Coat. Technol..

[86] Laleh M., Haghdadi N., Hughes A.E., Primig S., Tan M.Y. (2022). Enhancing the repassivation ability and localised corrosion resistance of an additively manufactured duplex stainless steel by post-processing heat treatment. Corros. Sci..

[87] Fathi P., Mohammadi M., Duan X., Nasiri A.M. (2019). Effects of Surface Finishing Procedures on Corrosion Behavior of DMLS-AlSi10Mg_200C Alloy Versus Die-Cast A360.1 Aluminum. JOM.

[88] Zeng F.-L., Wei Z.-L., Li J.-F., Li C.-X., Tan X., Zhang Z., Zheng Z.-Q. (2011). Corrosion mechanism associated with Mg2Si and Si particles in Al–Mg–Si alloys. Trans. Nonferrous Met. Soc. China.

[89] Revilla R.I., De Graeve I. (2018). Influence of Si Content on the Microstructure and Corrosion Behavior of Additive Manufactured Al-Si Alloys. J. Electrochem. Soc..

[90] Gu X.-H., Zhang J.-X., Fan X.-L., Zhang L.-C. (2019). Corrosion Behavior of Selective Laser Melted AlSi10Mg Alloy in NaCl Solution and Its Dependence on Heat Treatment. Acta Met. Sin. Engl. Lett..

[91] Gu X., Zhang J., Fan X., Dai N., Xiao Y., Zhang L.-C. (2019). Abnormal corrosion behavior of selective laser melted AlSi10Mg alloy induced by heat treatment at 300 °C. J. Alloy. Compd..

[92] Berlanga-Labari C., Biezma-Moraleda M.V., Rivero P.J. (2020). Corrosion of Cast Aluminum Alloys: A Review. Metals.

[93] Liu D., Atkinson H.V., Kapranos P., Jirattiticharoean W., Jones H. (2003). Microstructural evolution and tensile mechanical properties of thixoformed high performance aluminium alloys. Mater. Sci. Eng. A.

[94] Dutta B., Froes F.H.S. (2017). The Additive Manufacturing (AM) of titanium alloys. Met. Powder Rep..

[95] Laquai R., Müller B.R., Kasperovich G., Haubrich J., Requena G., Bruno G. (2018). X-ray refraction distinguishes unprocessed powder from empty pores in selective laser melting Ti-6Al-4V. Mater. Res. Lett..

[96] Zhang L.-C., Liu Y., Li S., Hao Y. (2018). Additive Manufacturing of Titanium Alloys by Electron Beam Melting: A Review. Adv. Eng. Mater..

[97] Bermingham M., McDonald S., Nogita K., John D.S., Dargusch M. (2008). Effects of boron on microstructure in cast titanium alloys. Scr. Mater..

[98] Chiu T.-M., Mahmoudi M., Dai W., Elwany A., Liang H., Castaneda H. (2018). Corrosion assessment of Ti-6Al-4V fabricated using laser powder-bed fusion additive manufacturing. Electrochim. Acta.

[99] Mwamba I.A., Cornish L.A., Van der Lingen E. (2014). Effect of platinum group metal addition on microstructure and corrosion behaviour of Ti–47·5 at-%Al. Corros. Eng. Sci. Technol..

[100] Lo K.H., Kwok C.T., Chan W.K., Kuan H.C., Lai K.K., Wang K.Y. (2016). Duplex Stainless Steels. Encyclopedia of Iron, Steel, and Their Alloys.

[101] Alvarez-Armas I., Degallaix-Moreuil S. (2009). Duplex Stainless Steels.

[102] Nilsson J.O., Chai G. (1997). The physical metallurgy of duplex stainless steels. Proc. Duplex Stainl. Steel.

[103] Llorca-Isern N., López-Luque H., López-Jiménez I., Biezma M.V. (2016). Identification of sigma and chi phases in duplex stainless steels. Mater. Charact..

[104] Biserova-Tahchieva A., Chatterjee D., van Helvoort A.T., Llorca-Isern N., Cabrera J.M. (2022). Effect of the nanostructuring by high-pressure torsion process on the secondary phase precipitation in UNS S32750 Superduplex stainless steel. Mater. Charact..

[105] Zhao Y., Wang Y., Tang S., Zhang W., Liu Z. (2019). Edge cracking prevention in 2507 super duplex stainless steel by twin-roll strip casting and its microstructure and properties. J. Mater. Process. Technol..

[106] Tavares S., Pardal J., Almeida B., Mendes M., Freire J., Vidal A. (2018). Failure of superduplex stainless steel flange due to inadequate microstructure and fabrication process. Eng. Fail. Anal..

[107] Sun Z.J., Tan X.P., Tor S.B., Yeong W.Y. (2016). Selective laser melting of stainless steel 316L with low porosity and high build rates. Mater. Des..

[108] Wang G., Liu Q., Rao H., Liu H., Qiu C. (2020). Influence of porosity and microstructure on mechanical and corrosion properties of a selectively laser melted stainless steel. J. Alloy. Compd..

[109] Eriksson M., Lervåg M., Sørensen C., Robertstad A., Brønstad B.M., Nyhus B., Aune R., Ren X., Akselsen O. (2018). Additive manufacture of superduplex stainless steel using WAAM. MATEC Web Conf..

[110] A Hosseini V., Högström M., Hurtig K., Bermejo M.A.V., Stridh L.-E., Karlsson L. (2019). Wire-arc additive manufacturing of a duplex stainless steel: Thermal cycle analysis and microstructure characterization. Weld. World.

[111] Lervåg M., Sørensen C., Robertstad A., Brønstad B.M., Nyhus B., Eriksson M., Aune R., Ren X., Akselsen O.M., Bunaziv I. (2020). Additive Manufacturing with Superduplex Stainless Steel Wire by CMT Process. Metals.

[112] Hejripour F., Binesh F., Hebel M., Aidun D.K. (2019). Thermal modeling and characterization of wire arc additive manufactured duplex stainless steel. J. Mater. Process. Technol..

[113] Knezović N., Garašić I., Jurić I. (2020). Influence of the Interlayer Temperature on Structure and Properties of Wire and Arc Additive Manufactured Duplex Stainless Steel Product. Materials.

[114] Posch G., Chladil K., Chladil H. (2017). Material properties of CMT—Metal additive manufactured duplex stainless steel blade-like geometries. Weld. World.

[115] Zhang Y., Cheng F., Wu S. (2021). The microstructure and mechanical properties of duplex stainless steel components fabricated via flux-cored wire arc-additive manufacturing. J. Manuf. Process..

[116] Zhang Y., Cheng F., Wu S. (2020). Improvement of pitting corrosion resistance of wire arc additive manufactured duplex stainless steel through post-manufacturing heat-treatment. Mater. Charact..

[117] Tavares S., Pardal J., Lima L., Bastos I., Nascimento A., de Souza J. (2007). Characterization of microstructure, chemical composition, corrosion resistance and toughness of a multipass weld joint of superduplex stainless steel UNS S32750. Mater. Charact..

[118] Kannan A.R., Shanmugam N.S., Rajkumar V., Vishnukumar M. (2020). Insight into the microstructural features and corrosion properties of wire arc additive manufactured super duplex stainless steel (ER2594). Mater. Lett..

[119] Stützer J., Totzauer T., Wittig B., Zinke M., Jüttner S. (2019). GMAW Cold Wire Technology for Adjusting the Ferrite–Austenite Ratio of Wire and Arc Additive Manufactured Duplex Stainless Steel Components. Metals.

[120] Saeidi K., Kevetkova L., Lofaj F., Shen Z. (2016). Novel ferritic stainless steel formed by laser melting from duplex stainless steel powder with advanced mechanical properties and high ductility. Mater. Sci. Eng. A.

[121] Wittig B., Zinke M., Jüttner S. (2021). Influence of arc energy and filler metal composition on the microstructure in wire arc additive manufacturing of duplex stainless steels. Weld. World.

[122] Papula S., Song M., Pateras A., Chen X.-B., Brandt M., Easton M., Yagodzinskyy Y., Virkkunen I., Hänninen H. (2019). Selective Laser Melting of Duplex Stainless Steel 2205: Effect of Post-Processing Heat Treatment on Microstructure, Mechanical Properties, and Corrosion Resistance. Materials.

[123] Shang F., Chen X., Wang Z., Ji Z., Ming F., Ren S., Qu X. (2019). The Microstructure, Mechanical Properties, and Corrosion Resistance of UNS S32707 Hyper-Duplex Stainless Steel Processed by Selective Laser Melting. Metals.

[124] Zheng Z., Gao Y., Gui Y., Zhu M. (2012). Corrosion behaviour of nanocrystalline 304 stainless steel prepared by equal channel angular pressing. Corros. Sci..

[125] Davidson K., Singamneni S. (2016). Selective Laser Melting of Duplex Stainless Steel Powders: An Investigation. Mater. Manuf. Process..

[126] Hengsbach F., Koppa P., Duschik K., Holzweissig M.J., Burns M., Nellesen J., Tillmann W., Tröster T., Hoyer K.-P., Schaper M. (2017). Duplex stainless steel fabricated by selective laser melting—Microstructural and mechanical properties. Mater. Des..

[127] Kim S.-T., Jang S.-H., Lee I.-S., Park Y.-S. (2011). Effects of solution heat-treatment and nitrogen in shielding gas on the resistance to pitting corrosion of hyper duplex stainless steel welds. Corros. Sci..

[128] Zhang Z., Zhao H., Zhang H., Hu J., Jin J. (2017). Microstructure evolution and pitting corrosion behavior of UNS S32750 super duplex stainless steel welds after short-time heat treatment. Corros. Sci..

[129] Nemani A.V., Ghaffari M., Salahi S., Nasiri A. (2023). On the microstructural characteristics and corrosion performance of as-printed and heat-treated PH 13–8Mo martensitic stainless steel fabricated by wire arc additive manufacturing. Mater. Today Commun..

[130] Kumar P., Jain N.K., Jaiswal S., Gupta S. (2023). Development of Ti–Ta–Nb–Mo–Zr high entropy alloy by μ-plasma arc additive manufacturing process for knee implant applications and its biocompatibility evaluation. J. Mater. Res. Technol..

[131] Illing C., Bestic M., Ernst F. (2023). Additive Manufacturing: Corrosion Proofing by Infusion of Interstitial Solute—Exemplified for Alloy 22. Metals.

